# Reports on Cities, Towns, and Districts

**Published:** 1856-04

**Authors:** J. H. Houghton

**Affiliations:** Officer of Health.


### SANITARY AND SOCIAL SCIENCE.

### REPORTS OF CITIES, TOWNS, & DISTRICTS.

### REPORT ON THE SANITARY STATE OF THE TOWN OF DUDLEY.

By J. H. HOUGHTON, Esq., M.R.C.S., Officer of Health.

THE town of Dudley, the principal and centre of that numerous group of towns which are situated in the populous and important district known as the South Staffordshire Coal-field, is itself built in the county of Worcester, on the very edge of the county: the castle and castle grounds, which adjoin the town, and form one of the most interesting objects in the district, being situated in the county of Stafford.

A chain of hills, elevated far above all surrounding objects, runs in a direction very nearly from south-east to north-west from Rowley Regis to Sedgley Beacon through the parish. The south-eastern portion of this chain is formed by basaltic rocks thrown up by volcanic convulsion to a very considerable height; the north-western portion is formed by the Wenlock limestone of the Silurian system. The town itself is built on the spot where these two formations meet. The soil covering that part of the parish which lies on the limestone is very strong and clayey, whilst that which lies on the basalt is of a much lighter and more earthy nature. The substratum is formed of a deep bed of strong clay of great depth, quite impermeable, hence the place is cold and, except in seasons of excessive drought, damp.

The surface of the town itself is very undulating, and on the northern and southern sides of it the fall is great in every direction. From the town itself the parish extends principally to the south and west, and here are placed the populous and important hamlets of Netherton and Woodside, which together at the last census contained a population of 8,128 souls. These two hamlets are situated at a level very many feet below the level of the town.

It is computed that the range of hills now spoken of forms the highest inhabited ground in the kingdom; and their very great altitude is proved by the facts that no canal has been brought into or very near the town, in spite of its manufacturing importance, and that the tunnel which passes under the town, and by which the traffic is maintained between the immense works situated on the northern and southern sides of the town, is at a level of 150 feet below the level of the town.

There is a curious fact worthy of mention in reference to the elevated position and drainage of Dudley, viz., that the rain and drainage from the houses on the eastern side of the High-street flows into the Trent and so into the German Ocean, whilst that from the houses on the western side flows into the Stour, and thence by the Severn into the St. George's Channel. The level of the town varies from 500 to 600 feet above the sea, though there are parts in the parish which rise to 1,000 feet.

The tower of the old castle stands on an eminence very many feet above, and commanding a complete view of the whole of the town and of the surrounding country; and it is probable that there are few spots in England from which a more glorious prospect can be obtained than from the top of the keep of Dudley castle. Within a range of ten miles twelve important towns, including Birmingham and Wolverhampton, can be seen; and in the farther distance the eye can range over an expanse of 120 miles in diameter, and reach into nine counties of England and Wales, embracing Malvern, Hatterill, the Wrekin, Bardell hill in Leicestershire, and many other places of less note.

The increase of the population of Dudley has been rapid. At the census in 1801 it amounted to 10,107 souls; in 1831 to 23,043; and in 1851 to 37,954 souls. From that time to the present, the excess of births over deaths has been 4,154, so that making due allowance for the migratory habits of some of the people, the population is now at least 42,116.

The majority of this population is employed in mining operations, in the smelting and manufacturing of iron in its various branches, and in the manufacture of glass. The wages obtained by the workmen are always high (except in times of great commercial depression), and in many cases would appear fabulous to persons not acquainted with the district. Hence a plentiful supply of all the necessaries of life, including animal food and beer, is within the reach of most people, except when their own follies or vices prevent a proper appropriation of the money earned.

In 1851 there were 7,337 houses in Dudley; hence, roughly speaking, there are about five and three-fourths persons to each house.

In a town of Dudley's great antiquity (for its origin is traced back so far as the Conquest) and recent and rapid growth, a great diversity in the character of the houses will be naturally expected, and so we find it; some of the very oldest being wretched hovels unfit for human habitations; others of a more recent date being small, low, ill ventilated and ill contrived; and others, lately built, and specially since the plans have been under the inspection of the local board, being of a superior description.

The Metropolitan Association for improving the Dwellings of the Industrial Classes have lately established a branch in Dudley; have purchased an excellent piece of land and just completed a block of ten houses; and are contemplating an extension of their operations, by which it is to be hoped the dwellings of the working men will be still materially improved.

In the year 1851, an inquiry was instituted by the General Board of Health into the sanitary state of Dudley; and the result of that inquiry was embodied in an able and copious report by Mr. Lee, to which I am indebted for some of the facts in this article.

It will be obvious from the general description which I have given of the elevated position and undulating surface of the town and parish of Dudley and of the surrounding area, that it has natural sanitary advantages of a very high order; and that, with one drawback incidental to its elevation, viz., deficiency of water, it has every natural requisite for a good state of health and longevity; its undulating surface affording the greatest amount of natural drainage, and its great elevation exposing it most freely to the western and northern gales, and so creating every facility for unusual natural ventilation. The sequel will shew that but for these two great advantages, and most probably the high "*condition*" resulting from the great consumption of animal food and nutritious diet, the mortality of Dudley would have been unique in the history of sanitary science.

The report of Mr. Lee was based on statistical facts ranging over a period of ten years ending 1850. The facts are carefully tabulated, so as to contrast them with the facts accumulated from other sources, and in commenting on them, Mr. Lee observes: "In whatever respect these tables are compared, whether as to the mortality per 1,000 of the population, or the proportion of deaths of infants, or the proportion of deaths from epidemics, or the average age of all who have died, or the proportion of deaths at 1, 5, 15, or 20 years of age, it will be evident that the inhabitants of the more favoured districts live at least half as long again as the inhabitants of Dudley." Again, "in the more healthy districts, about 16 per cent. of the deaths, or one-sixth of the whole, are those of children under one year of age, but in Dudley the proportion of those dying is about one-third of all the deaths on an average of 10 years. Under 15 years of age, the proportion in the more healthy districts is 29·4 per cent., in Dudley it is 67; under 20, the deaths in the more healthy districts are 32·9 per cent., but in Dudley it is 69·8 of the whole. In Dudley, therefore, it appears that about as large a proportion of the entire population dies in the first year of life as in the more healthy districts die under 20 years, and that in Dudley only 3 out of every 10 human beings born survive to years of maturity, whilst in districts containing more than 1,000,000 population about two-thirds of all born grow up and become men and women. The accidents in the mines, which it is often alleged are sufficient to account for the high rate of mortality, increase it only from 26·24 in the 1,000 to 26·97; whilst the deaths from epidemics, which are the most preventible of all diseases, increase the mortality from 20·67 per 1,000 to 26·97 of all living. In the more healthy districts the average duration of life is 41 years, whilst in Dudley it is only 18 years and 4 months, or less than one half." The average duration of life for the whole kingdom is 29 years; for London, 29 years; for Bristol, 29 years and 3 months; and for Sunderland and Newcastle-on-Tyne, both mining districts, it is 24. Mr. Lee sums up by saying that "*in no town in England is the work of extermination of human life so successfully carried on as in Dudley.*"

TABLE

*Showing the number of deaths, and their causes, for each quarter in the years ending September* 1854 *and* 1855.

| Disease and Age. | Quarters ending 1853-4. | | | | | Quarters ending 1854-5. | | | | |
|---|---|---|---|---|---|---|---|---|---|---|
| | Dec. | Mar. | June. | Sept. | Total | Dec. | Mar. | June. | Sept. | Total |
| Fever: under 15...... | 22 | 12 | 11 | 10 | 55 | 11 | 5 | 5 | 10 | 31 |
| „ over 15 ...... | 9 | 6 | 2 | 1 | 18 | 7 | 10 | 6 | 5 | 28 |
| Scarlet fever: under 15 | 84 | 36 | 15 | 13 | 148 | 10 | 6 | 3 | 1 | 20 |
| „ over 15 . | 1 | 1 | — | — | 2 | — | — | — | — | — |
| Measles: under 15.... | — | 9 | 19 | 6 | 34 | 13 | — | — | 7 | 20 |
| „ over 15 .... | — | — | — | 1 | 1 | — | — | — | — | — |
| Diarrhœa: under 15 .. | 23 | 21 | 18 | 34 | 96 | 33 | 14 | 7 | 11 | 65 |
| „ over 15.... | 6 | 2 | 1 | 9 | 18 | 10 | 6 | 1 | 7 | 24 |
| Cholera: under 15 .... | — | — | — | — | — | 28 | 1 | — | — | 29 |
| „ over 15 .... | — | — | — | — | — | 29 | 4 | — | — | 33 |
| Hooping cough: und. 15 | — | 7 | 1 | — | 8 | — | — | — | — | — |
| Other causes: under 15 | 148 | 178 | 106 | 117 | 549 | 123 | 213 | 111 | 74 | 521 |
| „ over 15 . | 99 | 113 | 84 | 75 | 371 | 101 | 171 | 88 | 93 | 453 |
| Total .......... | 392 | 385 | 257 | 266 | 1300 | 365 | 430 | 221 | 208 | 1224 |

I forbear to comment on facts so striking, lest in doing so I should weaken the force of them; but I rather proceed to develope the causes of this sad state of things.

The hasty sketch which I have given of the situation and contour of the spot on which Dudley is built and of the country which surrounds it, together with the fact that there is not any trade carried on in the place injurious to health, but on the contrary, that the occupations of the people are rather conducive to health, from their being carried on in the open air or nearly so, from their giving full development to the system, and from their affording wages sufficient to command a supply of good animal food, would lead, *primâ facie*, to a very different conclusion to that which statistics detail. In the absence, then, of natural causes, or of causes arising from occupation, we must look to causes of "man's creation"; in other words, to causes arising from breach of sanitary laws, and in them we shall find an ample explanation of the evils.

Up to July 1852, when the Public Health Act was first applied to Dudley, it may be said that the town and parish of Dudley were absolutely destitute of any sanitary care, sanitary powers, or sanitary knowledge, and a very strong impression generally prevailed that Dudley was an unusually healthy place, an impression which the unanswerable facts brought out by Mr. Lee, which facts have been amply corroborated by those which it has been my duty to bring forward, have failed to eradicate from the minds of some persons of considerable general intelligence, and some of great wealth and influence; but though there has been a good deal of criticism and mere assertion, there has not been any attempt to meet facts by facts, or to give any explanation of the facts consistent with the views of those who affect to disbelieve them.

I have not any records by which we may judge of the relative preponderance of each zymotic disease over a period of years, but the annexed table will show it for the two years ending September 1855. It will be observed that the table has been prepared simply to show the grand division between preventible and non-preventible disease, with the former of which at present we are alone concerned. Under the head of "other causes" are included marasmus, tabes, and infantile convulsions, each of which, I am very much disposed to believe, is practically as preventible as fever or cholera. A large number of deaths arise from these three causes in Dudley; in the year ending September 1855, one hundred and two deaths were registered from infantile convulsions alone, or exactly one in twelve of the whole deaths. The aspect of the table would be sadly changed if the deaths so caused were taken from "other causes" and added to the list above.

An examination of the table at once shows, that in the year ending September 1854, there were 380 deaths from zymotic diseases and 920 from other causes, the proportion being 1 zymotic death to 2·421 of other deaths. In the year ending September 1855, the zymotic deaths were 250, and the deaths from other causes 974; being in the proportion of 1 to 3·896. In the two years, the zymotic deaths were 630, and the deaths from other causes 1,844; the proportions being as 1 to 3·006. Of the total of these zymotic deaths, 506 were under, and 124 over, fifteen years of age; the proportions of deaths under fifteen being to those over fifteen years, in the ratio of 4·08 to 1.

The cholera alone caused a greater proportion of deaths in adults than it did in children. It will also be seen, that the mortality for two years from each preventible disease stands in the following order:

| | Persons. |
|---|---|
| Diarrhœa in the two years carried off | 203 |
| Scarlet fever | 170 |
| Fever (in its extended sense) | 132 |
| Cholera | 62 |
| Measles | 55 |
| Hooping cough | 8 |

There has been also a gradual but persistent decrease of zymotic deaths in every quarter for the last two years (except the cholera quarter); which happy result is attributable, I believe, partly to the proper sanitary supervision of the town which commenced then, and partly to a general decrease of zymotic diseases.

There is one most important fact shown in the table, which must not escape observation; viz., that in the year preceding that in which the cholera appeared in Dudley, the number of deaths from zymotic diseases was greater than in the year in which the cholera prevailed, in the proportion of 1·520 to 1, or rather over 50 per cent.; affording additional proof that cholera is always preceded by a great excess of zymotic diseases, and leading one almost to believe that there is some influence pervading nature, which, like a thunderstorm, goes on increasing in intensity as it proceeds, developing first fever and diarrhœa, and then cholera; after which it seems to have exhausted itself, and to have left us free from zymotic influences for a time. The fact also proves that we have less to fear in reality from the much dreaded cholera, than we have from fever and diarrhœa; and shows the absolute absurdity of sanitary activity only when its approach is expected, and sanitary apathy and indifference whilst fever is quietly but surely doing its work of destruction; in fact, it proves that sanitary vigilance should be unceasing.

After the ordinary zymotic diseases, and probably before them in point of number of persons attacked, come the diseases of the digestive organs, which the records of the dispensary prove to be very numerous. It will be remembered that the late Dr. Prout was of opinion that many of these diseases were of a malarious origin.

I may just allude here to the great prevalence of stone in Dudley, and the South Staffordshire Coal-field generally. Five operations for lithotomy have taken place within the last six months, in the practice of the Dudley Dispensary.

In endeavouring to develope the causes of the defective sanitary condition of Dudley, I will speak of—1. Drainage; 2. Water supply; 3. Hill-side houses; 4. Privy accommodation; 5. Pigs; 6. Overcrowding.

1. *Drainage.* The drainage of the town divides itself into the underground and surface. In speaking of the underground drainage, it is necessary to premise that my observations refer only to the parishes of St. Thomas and St. Edmond, containing together a population of 22,236 souls; the other three parishes of St. Andrew, St. James, and St. John, containing at the last census a population of 15,718 persons, being destitute of any attempt whatever at artificial drainage.

The remarks on the underground drainage will be easily summed up. The total length of roads in Dudley parish is thirty-six miles; of which twelve miles are turnpike roads, seven miles town streets; and the aggregate of all the public sewers little more than one mile in length. The evidence of Mr. Bateman, the town surveyor, given in 1851, goes to prove that most of these were nearly filled; and consequently, as Mr. Lee observes, "it may fairly be pronounced that Dudley is in a worse position than if no underground drainage existed." It is satisfactory to be able to state that the remedy for this great evil is close at hand. Admirable plans of the parish have been made by Mr. H. C. Roper, and approved by the General Board of Health. The sections are now in progress; and it is expected that the contracts for the new drainage will be out this spring.

The superficial drainage of some parts of the town is tolerably good; but there have sprung up within the last few years, in Dudley, hamlets, containing an aggregate of about ten thousand souls, in which there has not been any attempt whatever at the formation of roads, or of even superficial drainage. The streets have been laid out by the proprietors, without any controlling power, the traffic, which has been absolutely necessary, has been carried on over the deep clay, and so the surface has been thrown into every shape; here a pool of water, there a hillock; the refuse from the houses, animal and vegetable, with the excrements of the children, is constantly thrown into them, and lie festering on the surface, and the emanations from them, when the summer's sun is causing the evaporation of the moisture, may be conceived. The Local Board are at present engaged in endeavouring to find a remedy for this glaring evil, which, however, is one of very great difficulty, from the magnitude of the evil, and the great expense which the remedy must necessarily cause, whether *that* fall on the Board or on the owners of the property.

2. *Water Supply.* The state of the water supply will be best illustrated by an appeal to statistics, and to a few other facts which have passed under my observation. At the last census, the parish contained 7,337 houses; and by a return made by the manager of the Water Works Company, in the same year, it appears that 1,800 houses only are supplied with water by the Company, or 1 in 4·761 of the whole; but the Company's pipes are not carried into St. John's or St. Andrew's parishes at all, so that in these two parishes there was then 2,548 houses, containing 13,172 persons, destitute of water, so far as the Company is concerned, and this number has been greatly increased in the last five years by the immense increase of building which has taken place in St. John's district.

At the time referred to, the Company, in dry weather, had not half enough water for the supply of those houses to which their pipes were laid; and I have myself, *whilst living in the High Street, the principal street in the town, been compelled to go out without having my gig washed, because there was not water to wash it with. I have begged water for domestic use from my neighbours, and returned the compliment to them when I had an opportunity.*

My sole supply was from the Company, and the water was then laid on only every second, and sometimes every third day. What the condition of the poor (who had not the use of the pipes) was, must be imagined.

Since that time, the operations and resources of the Company have been very materially extended, and now the supply to their customers is plentiful, and tolerably good.

Besides the Water Works, the town has not any source of supply but the rain water and ordinary pumps, and the latter, in consequence of the mining operations, are an uncertain source of supply. In the three districts to which the pipes are conveyed, the proportion of houses supplied to those not supplied by the Company is 1 to 2·660; but it is certain that the majority of the houses supplied are inhabited by persons who can well afford to pay for it; hence the supply to the poor must necessarily be, as we find it by experience, very limited.

(*To be continued.*)

---

### SANITARY CONDITION OF PADDINGTON.

The parish of Paddington, situated at the western extremity of the metropolis, is bounded on the north-west by the open country, on the south by Kensington Gardens and Hyde Park, on the north-east by the parish of Marylebone and Notting Hill. It possesses many advantages, receiving from the west an atmosphere fresh from the country. The parish is divided in the Registrar-General's division into two sub-districts, St. Mary's lying to the north, St. John's lying to the south. By the late act, it is divided into four wards. Its superficial area is 1277 acres, its proportional area to that of the whole metropolis being as 1 to 61·81.

*The Geological Nature* of the superficial strata upon which Paddington is built varies. To the east of a line drawn from the canal bridge at Maida Hill to the head of the Serpentine, the soil on the surface is a mixture of gravel and sand. To the west of this line, the surface soil is a stiff clay of the London basin, with the exception of a portion of Kensington Gardens lying within the parish, and a small portion extending from hence across to the Uxbridge Road. These parts are gravel and sand.

*The Mean Elevation* of Paddington above Trinity high water mark is seventy-six feet, only four other parishes in London being higher, viz., Hampstead, Marylebone, Islington, and St. Pancras. The mean elevation of the sub-districts varies, that of St. Mary's being eighty-two feet, that of St. John's seventy-six.

*Drainage.* A small portion of the parish (a part of Kensal New Town) is drained into the Counter's Creek sewer, which discharges into the Thames on the south-east side of

Cremorne Gardens. The remaining sewers run into the common trunk, the Ranelagh sewer, which, commencing at Kilburn bridge, runs in a southerly direction through the parish, and discharges into the Thames on the south-east side of Chelsea Hospital. It has several branches, the chief of which commences in Grove End Road, extending thence by Lisson Grove, New Road, Grand Junction Road, and Albion Street, to a tumbling bay, where it joins the main sewer in Uxbridge Road. There is reason to believe that the calibre of this channel is not at all times sufficient to carry freely off the body of water which rushes through it.

*The Water Supply* of Paddington is from the Grand Junction Company, which now has its works above Hampton. This water is, we believe, identical in character with that of the West Middlesex, of which we gave an analysis in our last number. It is, taking it all in all, a good water.

*Houses and Streets*. The sub-district of St. Mary's contained, in 1851, 2557 houses, that of St. John's 4184. The new houses built in St. Mary's have in many cases been of an inferior class, and liable to epidemic visitations. The open spaces of the parish are numerous, the streets wide, and there are many squares. The surface of ground covered by open water is considerable—an objectionable sanitary arrangement. The average annual value of houses was £64 in the year 1851. In several streets, the houses are small, ill ventilated, closely packed, and inefficiently drained. The mortality of these streets is high.

*Population*. The population in 1851 was 46,305, giving an increase of 21,132 for the preceding ten years. The rate of increase was greatest in St. Mary's. By the same ratio of increase, the total is possibly now 54,000. Compared with a uniform distribution of the London population, there is in St. Mary's, Paddington, 1 person to every 242 square yards, and in St. John's 1 to every 75 square yards.

*Burial-grounds*. There are two graveyards within or close to the limits of the parish. Both are closed.

*Mortality*. The average annual death rate for a period of seven years, viz., from 1848-1854, was about 18·65 per 1000, being much below that of most other London districts. The highest mortality in the Paddington sub-districts, when compared with population, is in St. Mary's, being in this one 21·24 per 1000, while in St. John's it was 17·16. Deaths from epidemic causes are highest in St. Mary's. The deaths in seven years were, in St. Mary's, from all causes 2591, from epidemics 495; in St. John's, from all causes 3511, from epidemics 649. The streets in which deaths from epidemic causes are most marked, are, in St. John's—Star Street, Market Street, Praed Street, South Wharf Road, Caroline Place, Stanley Street, Cambridge Place, Conduit Street, Union Place, Elmes Lane and Place, Brooke's Mews, Tichborne Street, Moscow Road, Polygon Mews, Sale Street, and Charles Mews; in St. Mary's—North Wharf Road, Hampden Street, Church Place, Brindley Street, Workhouse, Hall Park and Place, Pickering Place, White Lion Place, Kensal Road, Dudley Street, Welling's Place, and Waverly Road; these streets differ widely from each other. The North Wharf Road, where 39 deaths from epidemic diseases have occurred in the seven years, is situated on the north side of the canal basin, and its houses are low, ill-ventilated, and subjected to the effluvia from the stagnant water of the canal and the refuse lying near it. A similar condition obtains in South Wharf Road. In Star and Market Streets, where the mortality is highest of all from epidemics, there are a large number of dwellings with little space behind, and with ventilation exceedingly defective. Of the different epidemics during the seven years, scarlatina was most fatal, but typhus and other forms run high and are on the increase. Cholera destroyed 31 persons within the parish in 1849, and 77 in 1854. On both occasions the disease visited in many instances the same quarters of the parish, the greatest mortality occurring in Elmes Lane. Paddington holds a better sanitary position than many other metropolitan districts. But it admits of great improvement, and indicates, as yet, far too prominently the evils of low elevation, bad ventilation, indifferent drainage, and the other nursing mothers of disease and death.

The above facts are all abstracted from an excellent treatise bearing the title we have transcribed, by Dr. Graily Hewitt, to whom we offer public thanks for so useful an addition to sanitary literature.

---

### MORTALITY OF THE PARISH OF CROYDON,

*For the Years ending December* 31*st, from* 1848 *to* 1855, *inclusive.*

It is very satisfactory to find generally, that a large proportion of the zymotic class of diseases are confined to districts without drainage or water supply,—including the northern and eastern portions of the parish, viz., part of Croydon Common, Norwood, and Shirley. The total number of deaths in 1855 from all causes is 508, which is less by 112 than would have occurred at the average rate of mortality per annum for the last seven years, allowing for increase of population. The births for the year have been 364 females and 383 males; total, 747. The total number of houses in the parish is now 4,287. Of this number, there are in the special district, 3,296; and of these, supplied with water, 2,046; shewing still without water supply, 1,250; and probably 200 or 300 above that number in the special district unconnected with the drains. Notices for 178 new houses have been received and approved in the year 1855. The comparatively large number of houses for the year 1855, arises from the fact of their being taken from the rate books, and the new houses have not hitherto been included. For the same reason the vacant houses shew a like disproportion —all houses now in course of erection being included in that return.

POPULATION TABLE.

| Year. | Population by Census. | Estimated Population. | Number of Houses. | Houses Vacant and Building. | Births for Year. | | |
|---|---|---|---|---|---|---|---|
| | | | | | F. | M. | Total. |
| 1841 | 16,712 | ” | ” | ” | ” | ” | ” |
| 1848 | ” | 19,380 | 3060 | 280 | 262 | 260 | 522 |
| 1849 | ” | ” | 3107 | 283 | 290 | 297 | 587 |
| 1850 | ” | ” | 3171 | 170 | 298 | 267 | 565 |
| 1851 | 20,355 | ” | 3234 | 175 | 291 | 312 | 603 |
| 1852 | ” | ” | 3345 | 201 | 287 | 331 | 618 |
| 1853 | ” | ” | 3622 | 177 | 368 | 347 | 715 |
| 1854 | ” | ” | 3721 | 258 | 233 | 374 | 707 |
| 1855 | ” | 25,837 | 4287 | 344 | 364 | 383 | 747 |

The average mortality of Croydon, from all causes of disease and at all ages, for the seven years ending 1854, was 506 for the whole population, or 2·426 per cent. The number of deaths in the year 1855 was 508, or 1·975 per cent. The deaths at different ages, during the seven years above-named, gave the following average.

| Date. | Under 2 years | 2 to 10 years. | 10 to 20 years. | 20 to 40 years. | 40 to 60 years. | 60 to 80 years. | 80 and upwards | Total. |
|---|---|---|---|---|---|---|---|---|
| 7 yrs. end. 1854 | 119·6 | 93·2 | 39·3 | 73·1 | 68·6 | 86·1 | 25·3 | 506 |
| For 1855...... | 137 | 57 | 33 | 72 | 80 | 101 | 28 | 508 |

The mean temperature of the seven years was 49·39; rainfall, 24 inches. The mean temperature of 1855 was 48·24; rainfall, 23 inches. In the seven years, among the causes of death, lung-diseases hold a high figure. Fever is at the head of the zymotic class, while deaths by accident and suicide average 10 per year. The result of the above tables for the year 1855 is most satisfactory; for, although the per-centage is still higher than the Registrar General's standard for a healthy community, it may be considered a good return for so compound a parish; since, in addition to a very densely populated town district, there are in Croydon (besides other large establishments) the Central London District School, with an average of 1,050 children—shewing 27 deaths for the year; the Union house, with a weekly average of 213—shewing 49 deaths for the year; and Croydon barracks, with an average of 433 inmates—shewing 20 deaths for the year. Sixteen of 30 deaths from fever occurred in the undrained districts. There is a decrease in all diseases of the zymotic or endemic class, and an increase in those diseases only which are especially affected by the low average temperature of the year, and inimical to infancy and old age. (*Compiled from a report by* E. Westall, Esq.)

---

### ALLOTMENTS FOR THE LABOURING POOR AT SOUTHAMPTON.

[Dr. Bullar, of Southampton, has kindly sent to the Journal of Public Health the following account of the principle of an allotment system at Southampton, abridged from a Report of the Allotment Committee.]

The Report itself is published in the hope that it may induce others, who live in large towns, to undertake the management of allotments for the labouring poor on a similar plan, which consists in a committee renting the ground, having it divided, and letting it as allotments under their regulations; not as an act of charity, but by hiring the land at its market price and charging the allottees sufficient to cover all expenses.

In country places, the allotment system has been carried out chiefly by the landowners themselves; but where there is no resident proprietor who is willing to take the trouble, as in large towns, the present plan will be found feasible, and of great benefit to the industry, comfort, and health of the labouring poor.

In consequence of the expressed wish of many labouring men for allotments, and the refusal of the owners of land to let any quantity to poor men who could not be responsible for a large sum, a Working Committee of three was formed.

The Committee hired a field of eighteen acres, and had it divided by a surveyor into 146 allotments of twenty rods each, or eight allotments to an acre, with convenient paths.

The first year (1850) the committee charged the allottees 6*d.* a rod, or 10*s.* an allotment, which was at the rate of £4 an acre.

In three years the bad debts amounted to only £8 : 13 : 0, and £222 : 3 : 0 was received, leaving a balance of £10 : 14 : 1 in hand after paying all expenses.

The committee cannot but think this statement will be acknowledged as most satisfactory, in regard to the payment of rent by so large a body of allottees taken indiscriminately in a large town where superintendence by the committee was out of the question; and that it shows the poor to be excellent tenants, when the rent is diligently looked after by a zealous and honest collector.

In 1852, the Rev. J. C. Wigram, Archdeacon of Winchester, was appointed Rector of St. Mary's parish, which contains 21,220 inhabitants, a large proportion of whom are of the labouring classes. From the experience of the committee, Archdeacon Wigram, with the full sanction and desire of the Bishop of Winchester, decided to let on the same plan twenty acres of glebe land conveniently contiguous to the houses of the poor. The same committee, with the Archdeacon as chairman, undertook the management, renting the land at the full price at which it had been let to market gardeners and graziers. It was partly arable and partly grass land separated by ditches, with the remains of an abandoned canal running through it, so that the committee were at a considerable expense in levelling, fencing, and draining it. It was next surveyed and divided into 162 allotments of twenty rods each (a few having more or less, according to the shape of the land), and with paths broad enough to admit small carts. The committee, besides paying all rates and other expenses, agreed to give £6 an acre for six acres, and £5 an acre for fourteen acres, the full market price of the land; and as they did not wish to make in any respect a charity, they found that they could not cover their expenses and risks at a less charge than 11*d.* a rod, or 18*s.* 4*d.* an allotment, except for a few allotments where the land was inferior, and which were charged at 9*d.* a rod. Eleven-pence a rod is at the rate of £7 : 6 : 8 an acre. In this instance the allottees were also taken indiscriminately as they applied, and were not restricted to parishioners.

At the end of three years £418 : 11 : 0 had been received.

| | | | | |
|---|---|---|---|---|
| The bad debts in | 1851-2 | were | £3 : 13 : 8 |
| „ „ | 1852-3 | „ | £10 : 13 : 4 |
| „ „ | 1853-4 | „ | £2 : 15 : 0 |

that is only £17 : 2 : 0 in three years.

The committee have paid the collector at the rate of one shilling a year for each allotment. For this he takes the names of the applicants, procures their signatures to the cards, collects the rents, and attends the meetings of the committee when required.

The sanitary advantages of allotments to the poor of large towns must be very considerable. They have, as a rule, no available gardens, and cannot afford to buy in the market or of green-grocers that due supply of fresh vegetables which they require. These allotments furnish with fresh vegetables and with good potatoes 308 families; that is, above 1600 persons. To those engaged in sedentary occupations in close courts, alleys, and lanes, with no inducement for taking exercise, the cultivation of allotments at some little distance from their homes is most desirable, as it gives them the most efficient kind of exercise in the open air, with a good object.

There is another sanitary advantage in the allotments affording an immediate and useful outlet for manure from the pigsties and stables, and for the refuse of the houses of the poor. It is remarked that the allottees "farm high", and consequently make the most of that which is so much wasted in this country. Indeed, an allotment is often the poor man's bank, in which he invests much of his savings. The value of allotments is also great, inasmuch as they satisfy one of the strongest and most wholesome desires of the labouring poor—the wish to possess land and to cultivate it for themselves.

In this town there are numbers of working men, such as those employed in the docks in unloading ships, bricklayers, masons, painters, and common labourers, who are never fully employed; many will tell you that they are unemployed three, four, and even six months out of the twelve. To supply such with the means of profitable and healthy occupation, and such as they like, in their tedious compulsory leisure, which they would otherwise spend in idleness, or in frequenting beershops, is a service to them, morally as well as economically. Moreover, the working men in these parts expect and require this kind of assistance from those who are socially above them; they receive gratefully and value greatly such disinterested attempts to improve their condition.

